# Blue Photosensitizer, Red Light, Clear Results: An Integrative Review of the Adjunctive Periodontal Treatment with Methylene Blue in Antimicrobial Photodynamic Therapy

**DOI:** 10.3390/dj13070289

**Published:** 2025-06-26

**Authors:** Higor Henrique Carvalho Oliveira, Gabriela Moura Chicrala-Toyoshima, Carla Andreotti Damante, Rafael Ferreira

**Affiliations:** 1 Faculdade de Odontologia, Universidade Federal de Mato Grosso do Sul, Campo Grande 79070-900, Brazil; higor_carvalho@ufms.br; 2 Discipline of Stomatology, Faculdade de Odontologia, Universidade Federal de Mato Grosso do Sul, Campo Grande 79070-900, Brazil; gabriela.chicrala@ufms.br; 3 Discipline of Periodontics, Faculdade de Odontologia de Bauru, Universidade de São Paulo, Bauru 17012-901, Brazil; 4 Discipline of Periodontics, Faculdade de Odontologia, Universidade Federal de Mato Grosso do Sul, Campo Grande 79070-900, Brazil; rafael_ferreira@ufms.br

**Keywords:** periodontal disease, photodynamic therapy, periodontitis, methylene blue, revision, adjunctive therapy

## Abstract

The adjunctive use of antimicrobial photodynamic therapy (aPDT) has been investigated as a promising approach to enhance periodontal therapy. Methylene blue (MB) is the most commonly used photosensitizer due to its favorable characteristics, including a neutral pH and an absorption peak at 660 nm. Due to the considerable heterogeneity among studies and the lack of well-established clinical protocols, this study aims to conduct an integrative review to highlight the effects of MB-mediated aPDT as an adjunct to periodontal treatment. The inclusion criteria were randomized clinical trials that used MB as the PS, published between 2009 and 2024, with a minimum follow-up of three months. Studies included patients with periodontitis treated with SRP alone or in combination with aPDT. Of the 237 studies initially identified, 23 met the eligibility criteria and were included in this integrative review. The risk of bias was evaluated using the Cochrane criteria for randomized controlled trials. Although the included studies reported heterogeneous clinical outcomes, a general trend toward improvement in key periodontal parameters—probing depth, bleeding on probing, clinical attachment level, and plaque index—was observed when MB-mediated aPDT was used as an adjunct to conventional periodontal treatment. However, substantial variability in clinical protocols—including differences in photosensitizer concentration, type of light source, irradiation time, and frequency of application—limited the comparability of results across studies. Despite these methodological inconsistencies, current evidence suggests that MB-mediated aPDT holds promise as an adjunctive approach in periodontal therapy. Nevertheless, due to the clinical heterogeneity and the limited number of studies with long-term follow-up, its overall efficacy remains inconclusive. Further well-designed randomized controlled trials with standardized protocols and subgroup analyses are essential to validate its clinical relevance.

## 1. Introduction

The use of light-based therapies in periodontics has gained prominence as a favorable therapeutic approach, particularly through the application of low-level laser therapies [1]. The treatment of choice for periodontal disease remains oral hygiene instruction combined with scaling and root planing (SRP), aimed at the removal of biofilm, calculus, and contaminated cementum. The primary clinical goal is to achieve a probing depth (PD) of ≤4 mm without bleeding on probing (BOP) [2]. To enhance the clinical outcomes (PD, BOP Clinical Attachment Level-CAL and Plaque Index-PI) of SRP, various adjunctive therapies have been proposed [3]. In this context, the adjunctive use of antimicrobial photodynamic therapy (aPDT) may also be employed as an adjunct to SRP, contributing to the reduction in bacterial load and inflammation in periodontal tissues, while additionally promoting biostimulatory effects [4].

Currently, the scientific literature has increasingly highlighted the benefits of aPDT in both periodontics and implantology [1], demonstrating that this approach is effective and safe for a variety of clinical conditions. aPDT acts on multidrug-resistant microorganisms and, apparently, does not induce bacterial resistance [5].

In aPDT, the type of photosensitizer (PS) can significantly influence therapeutic outcomes. Among the available PSs, synthetic dyes such as methylene blue and toluidine blue are among the most used in clinical protocols [6]. Methylene blue (MB) is particularly prominent due to several advantageous characteristics, including its low cost, market availability, and favorable clinical performance [7]. Furthermore, MB possesses a neutral pH, a peak absorption at 660 nm, and is hydrophilic in nature [8].

Despite promising results, the literature still presents conflicting findings regarding the efficacy of aPDT as an adjunct to conventional periodontal therapy. Considering that MB is the most widely used PS and that its absorption peak (660 nm) corresponds to the emission wavelength of many commercially available lasers, this study aims to conduct an integrative review to highlight the effects of MB-mediated aPDT as an adjunct to periodontal treatment.

## 2. Materials and Methods

The present literature review was structured to answer the following question: “Does adjuvant aPDT using MB as a photosensitizer promote additional effects in periodontal treatment?”

### 2.1. Study Design

This review analyzed randomized clinical trials that investigated the effects of antimicrobial photodynamic therapy (aPDT), mediated by MB, in patients diagnosed with periodontitis (Stage 1 to 4). The objective was to conduct a literature review to investigate the controversial outcomes of antimicrobial photodynamic therapy mediated by methylene blue as an adjunctive treatment in periodontal therapy, highlighting its potential clinical benefits. Accordingly, the following primary clinical parameters were evaluated: Probing Depth (PD), Bleeding on Probing (BOP), Clinical Attachment Level (CAL), Plaque Index (PI), Gingival Index (GI), Gingival Recession (GR), and Gingival Level (GL). Secondary outcomes included microbiological and/or immunological analyses (when available). The classification of systemic conditions and deleterious habits was based on the original focus and objectives of the included studies, which specifically aimed to investigate periodontal therapy in these patient populations.

### 2.2. Eligibity Criteria

#### 2.2.1. Inclusion Criteria

Randomized clinical trials; studies that applied MB as photosensitizer; studies involving patients with periodontitis (stage 1 to 4), treated by adjuvant aPDT and accompanied for at least 3 months; full-text available studies; studies that included smokers; studies involving patients with furcation lesions and residual periodontal pockets; studies that evaluated periodontal clinical parameters and/or microbiological and/or immunological analysis (when available); studies involving patients with periodontitis and systemic diseases; studies in English language.

#### 2.2.2. Exclusion Criteria

Studies that used other photosensitizer than MB; animal or in vitro studies; studies that exclusively evaluated gingivitis; studies that evaluated peri-implantitis or periimplant mucositis; studies presenting insufficient or unclear information that hindered data extraction; absence of response from authors after e-mail requesting additional information.

### 2.3. Search Strategy

The research was conducted in the MEDLINE (PubMed) database using keywords combined with Boolean operators (OR, AND). All articles published between 2009 and August 2024 were included, with a restriction to publications in English. The search strategy involved the terms “periodontal disease and antimicrobial photodynamic therapy” and “periodontitis and antimicrobial photodynamic therapy,” with the selection of “clinical trial” as the article type. In addition to the electronic search, manual searches were also performed to complement the results obtained.

### 2.4. Study Selection

For the selection of studies, the titles and abstracts were initially evaluated, followed by a full-text assessment. The studies that met the criteria outlined in this review were then subjected to data acquisition and risk of bias analysis.

### 2.5. Data Collection Process

In the data collection process, two investigators (HHCO and RF) were responsible for the data collection and review. When necessary, the authors of the selected articles were contacted to clarify any doubts.

### 2.6. Risk of Bias

All the studies selected for this review underwent a risk of bias assessment. This assessment was conducted using the Cochrane Risk of Bias tool for randomized trials (RoB 2.0), which considers five domains: (D1) Randomization process and timing of participant identification or recruitment; (D2) Deviations from intended interventions; (D3) Missing outcome data; (D4) Outcome measurement; (D5) Selection of reported outcomes. Based on the analysis of these studies, they were classified as having high risk of bias (+), low risk of bias (−), or unclear risk of bias (!).

### 2.7. Data Analysis

For the data synthesis, the following information was collected from the selected articles: authors and year of publication, clinical parameters assessed, microbiological parameters assessed, study design type, number of patients involved in the research, the underlying disease of the patients involved, data related to the laser used, its dosimetry, the light source, whether local anesthesia was used for the procedure, whether pre-irradiation irrigation was performed, the concentration of the dye, the pre-irradiation time of the PS, the frequency of aPDT sessions, and the main results found. 

## 3. Results

**Figure 1:**
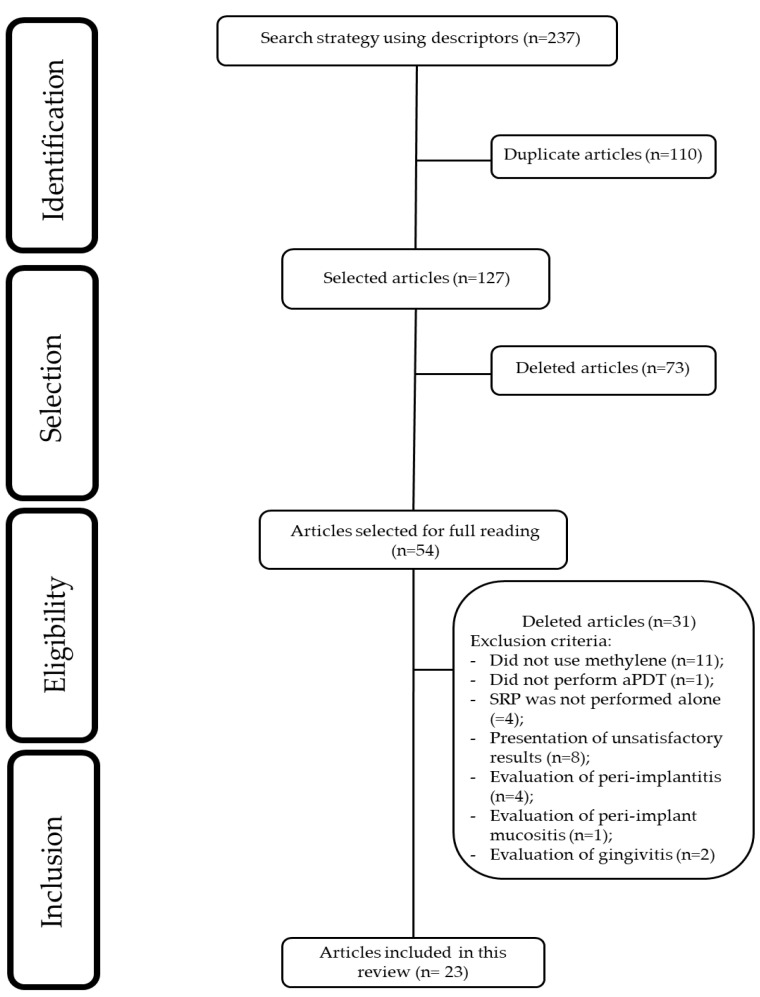
Flowchart of study selection.

As a result of the database search using the descriptors ‘periodontal disease and antimicrobial photodynamic therapy,’ 123 articles were retrieved, and with ‘periodontitis and antimicrobial photodynamic therapy,’ an additional 114 articles were identified. In total, 237 potentially relevant articles were found. Duplicate records (*n* = 110) were removed using the Rayyan platform. After screening titles and abstracts, 73 articles were excluded based on relevance, and 54 were selected for full-text assessment. Following a detailed evaluation, 30 articles were excluded with justification. Ultimately, 23 articles met the eligibility criteria and were included in this review. The selection process of the articles is illustrated in Figure 1.

### 3.1. Clinical Studies Included in This Review

The clinical trials selected for this integrative review addressed different patient profiles. Table 1 shows the quantitative distribution of these studies.

Table 2 provides a summary of the studies selected for this review. Table 3 presents the follow-up periods of the clinical trials. Table 4 presents the methods of randomization used in the studies. Table 5 displays the data on statistically significant differences between the test and control groups for each variable.

The studies included in this review employed either a split-mouth design (*n* = 9) [16,18,22,23,25,26,28,29,31] or a parallel-group design (*n* = 14) [9,10,11,12,13,14,15,17,19,20,21,24,27,30].

Additionally, only (*n* = 6) [10,14,17,27,28,31] studies evaluated microbiological parameters. One study assessed levels of *P. gingivalis* (Pg) and *P. intermedia* (Pi) [10], while another analyzed levels of *P. gingivalis* (Pg), *P. micra* (Pm), and *P. intermedia* (Pi) [17]. Levels of *P. gingivalis* (Pg), *A. actinomycetemcomitans* (Aa), *T. forsythia* (Tf), *T. denticola* (Td), *P. intermedia* (Pi), and *P. micra* (Pm) were evaluated in [27]. Other studies examined levels of *A. actinomycetemcomitans* (Aa), *P. gingivalis* (Pg), and *T. forsythia* (Tf) [14,28,31].

Local anesthesia was used in 11 studies [10,14,16,17,18,20,23,25,28,29,30]. In contrast, 6 studies did not use local anesthesia [11,15,19,21,22,26]. Its use was not reported in 5 studies [9,12,24,27,31], and in 1 study it was administered only if necessary [13].

Local irrigation after photosensitization was performed in 9 studies [11,14,18,20,21,24,25,28,29], whereas in 11 studies it was not performed [9,10,13,15,16,17,19,22,26,27,30]. In 3 studies, this information was not reported [12,23,31].

Regarding the pre-irradiation time, the studies were quite heterogeneous, with durations ranging from 10 s [13,15,30], 1 min [9,10,14,17,20,22,27,28,29], 2 min [16], 3 min [24,25], to 5 min [11,18,19,21,23,26,31]. Only one study did not report the pre-irradiation time used [12].

To complement the findings from clinical studies based on current scientific evidence, Table 6 presents the systematic reviews related to periodontal treatment using antimicrobial photodynamic therapy (aPDT) as an adjunctive approach. A noticeable heterogeneity is observed regarding the number of included studies, as well as the inclusion of animal studies within some of these reviews.

### 3.2. Risk of Bias Assessment

**Figure 2:**
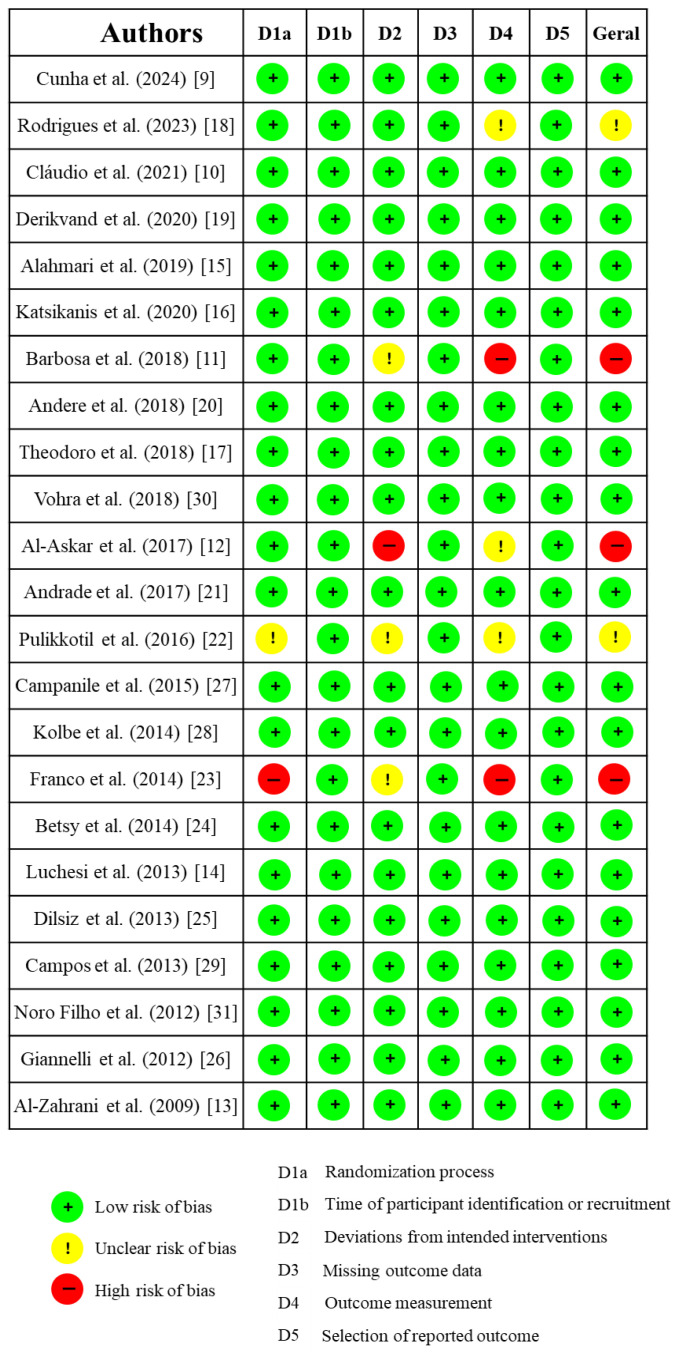
Risk of bias.

The risk of bias assessment identified 19 clinical trials as having a low risk of bias [9,10,13,14,15,16,17,19,20,21,24,25,26,27,28,29,30,31], as observed in Figure 2. In contrast, three studies were classified as having an unclear risk of bias [18,22], and three were considered to have a high risk of bias [11,12,23].

The studies deemed to have a high risk of bias received this classification primarily due to the absence of methodological details regarding randomization, which made it unclear whether adequate blinding of researchers was implemented [11,12,23]. In one of the studies categorized as having an unclear risk of bias, baseline characteristics of the participants were not sufficiently reported to determine potential differences between groups, and homogeneity among groups was not addressed [18]. Additionally, the randomization process was inadequately described in one of the included studies [22].

## 4. Discussion

This integrative literature review aimed to examine the efficacy of antimicrobial photodynamic therapy (aPDT), mediated by the MB photosensitizer, as an adjuvant in the treatment of periodontitis. Upon analyzing the selected articles, it was observed that most clinical studies demonstrated favorable outcomes for aPDT as an adjunct to scaling and root planing (SRP) in the non-surgical management of periodontal disease (PD), albeit often limited to a single assessed periodontal parameter [11,18,19,20,22,23,24,25,26,27,29,31]. However, several clinical trials reported no statistically significant differences between aPDT and the control group (SRP) for any of the evaluated clinical parameters [10,12,13,14,17,21,28].

Most articles in this review focused on the application of aPDT in chronic periodontitis treatment [10,11,12,13,14,15,16,17,18,19,20,21,23,30,31] or in residual pockets [27,28,29] among patients undergoing periodontal maintenance therapy. Notably, MB was consistently used as the photosensitizer (PS) in combination with 660–670 nm laser light, which corresponds to the optimal absorption spectrum of this PS.

It is crucial to emphasize that all included articles underwent rigorous risk-of-bias assessment, even within the context of an integrative literature review. The evaluation of the risk of bias in randomized clinical trials is paramount to ensure the reliability of reported findings. Studies with a high risk of bias may yield skewed conclusions, thereby compromising the integrity of the results and subsequent clinical decision-making. Furthermore, poor randomization and lack of masking may result in an overestimation of the intervention effects in randomized clinical trials [44].

The distribution of experimental designs among the reviewed studies was relatively homogeneous. The split-mouth model was employed in 9 studies [16,18,22,23,25,26,28,29,31] whereas the parallel-group design was utilized in 14 studies [9,10,11,12,13,14,15,17,19,20,21,24,27,30]. Split-mouth studies enrolled as few as 12 or 13 patients [29,31] or up to 26 patients [26]. In contrast, the largest parallel-group trials recruited 83 and 88 patients, respectively [15,24].

Regarding probing depth (PD) outcomes, several included studies reported PD reduction in patients treated with scaling and root planing (SRP) combined with aPDT (*n* = 9) [18,19,20,24,25,26,27,29,31]. Notably, only one clinical trial demonstrated PD reduction favoring the SRP-alone group [9], though this involved patients with diabetes, a population with distinct clinical considerations. Despite these promising findings for aPDT as an adjuvant therapy in PD reduction, it is critical to acknowledge that numerous other studies (*n* = 13) reported no statistically significant superior outcomes for aPDT [10,11,12,13,14,15,16,17,21,22,23,28,30]. The positive outcomes of the therapy, particularly regarding improvements in periodontal status (PS) and clinical attachment level (CAL), are highly relevant to its clinical applicability. Furthermore, these findings are essential to support the development of future therapeutic protocols.

Thus, while the data suggests potential benefits of aPDT, it is imperative to exercise caution in interpreting these results as conclusive evidence for its widespread clinical validation. Significant heterogeneity exists across studies, particularly regarding patient profiles (e.g., systemic conditions like diabetes), PS concentration, number of aPDT applications, and light dosimetry parameters. These methodological discrepancies underscore the need for standardized protocols and further high-quality randomized controlled trials to establish definitive conclusions.

Improvement in bleeding on probing (BOP) in patients who received aPDT as an adjunctive treatment was observed in several studies (*n* = 7) [11,22,23,24,26,29,31]. A common feature among most of these studies was the use of a split-mouth design (*n* = 5) [22,23,26,29,31]. Moreover, the majority of studies used a fiber-optic tip for intrapocket light delivery [23,24,26,29,31], which may favor the photodegradation reaction of the photosensitizer. However, there was considerable heterogeneity regarding photosensitizer concentration, pre-irradiation time, and number of applications, which introduces bias when evaluating the actual efficacy of this clinical parameter.

Furthermore, four studies reported a statistically significant improvement in clinical attachment level (CAL), with better outcomes observed in patients who received adjunctive aPDT [24,25,26,29]. These findings suggest that aPDT may contribute to additional improvements in CAL when compared to conventional treatment alone. However, this interpretation should be approached with caution due to variability in the aPDT application protocols and differences in patient characteristics across the studies. Such heterogeneity reflects inconsistencies in the outcomes, underscoring the need for further in-depth analysis.

This analysis also extends to other clinical parameters, such as plaque index (PI) and gingival recession (GR). Regarding PI, only a few studies (*n* = 2) reported improvement in this clinical parameter among patients treated with aPDT [11,23]. In contrast, other studies found lower plaque levels in patients treated with scaling and root planing (SRP) alone [15,16,30]. Similarly, studies assessing gingival recession (GR) were limited. Most of those that did analyze GR reported no statistically significant differences between groups [9,18,20,27,31].

In recent years, the potential of aPDT to secondarily promote periodontal healing has been widely discussed, particularly when a low-level laser is used as the light source. In animal studies, this effect has been reported by some authors [45,46]. Although certain clinical studies did not demonstrate significant improvements in clinical parameters, they showed favorable outcomes regarding inflammatory markers, such as a reduction in MMP-8 [47]. Additionally, one study reported a decrease in TNF-α levels in patients treated with aPDT [9]. There is also the possibility of using low-level laser therapy independently, either immediately after SRP or in alternating sessions with aPDT, to promote tissue repair. However, it is important to emphasize that this approach does not fall within the standard aPDT protocol.

In relation to repetition of applications, a study which applied between 4 and 10 sessions of aPDT suggests that increasing the number of aPDT applications may enhance clinical outcomes [26]. The highest number of aPDT sessions among all those included in this review resulted in improvements across all evaluated clinical parameters (probing depth [PD], bleeding on probing [BOP], and clinical attachment level [CAL]). It also employed a longer pre-irradiation time (5 min), compared to other studies. This methodology remains underexplored, particularly in studies assessing the effectiveness of methylene blue as a photosensitizer. A pioneering study highlighted the benefits of aPDT using the HELBO^®^ system in patients undergoing periodontal maintenance [48]. Five sessions of aPDT over a two-week period led to clinical improvements that persisted for up to six months post-treatment. However, even a single application of aPDT yielded positive results in clinical parameters (PD, BOP, CAL, and PI), despite a shorter pre-irradiation time of 3 min [24]. Therefore, the assumption that increasing the number of aPDT sessions will necessarily lead to better periodontal outcomes remains inconclusive and warrants further investigation through well-designed clinical trials.

Antimicrobial photodynamic therapy (aPDT) was applied twice a week for the treatment of residual pockets, resulting in a reduction in probing depth and C-reactive protein levels. These benefits were sustained for up to three months [27]. Additionally, the treatment resulted in the absence of residual pockets at the end of the study. All three articles included in this review that assessed residual pockets included patients diagnosed with periodontitis who had previously undergone supportive periodontal therapy prior to the experimental intervention [27,28,29]. Only two studies [27,29] demonstrated improvement in probing depth for patients who received aPDT. Improvements in bleeding on probing (BOP) and clinical attachment level (CAL) were also reported [29].

The use of aPDT in the treatment of furcation lesions did not demonstrate improvements in periodontal clinical parameters. A study [14] that focused on class II furcation lesions did not find specific clinical differences between the groups. Besides that, the researchers observed a reduction in *Porphyromonas gingivalis* (Pg.) and *Tannerella forsythia* (Tf.) bacteria during the 6-month follow-up. This was the only study included in this review that addressed furcation lesion treatment, and it is important to note that no biomaterial was used in the treatment.

In addition to surgical approaches, other alternatives have been proposed. A combination of high-intensity lasers was used to remove all epithelium from the periodontal pocket before aPDT [26]. This technique resulted in improvements in clinical parameters, with long-lasting effects observed up to one year after treatment.

There are still few publications on the use of aPDT in patients with compromised systemic conditions. In the case of patients with diabetes, aPDT was compared or combined with antibiotics, such as doxycycline. This combination did not result in improvements in periodontal clinical parameters, although a reduction in glycated hemoglobin was noted in the group that received antibiotics alone [13]. These results suggest that, while aPDT has potential, it may not be as effective when used alone in patients with diabetes, highlighting the need for further investigation in this area. In a study analyzing patients with type 1 diabetes, a significant reduction in TNF-α levels in crevicular fluid in the group treated with aPDT was observed [9]. Additionally, plaque index varied between patients with and without diabetes, with the diabetes group showing a higher plaque index. There were no statistically significant differences in probing depth between the diabetes and non-diabetes groups that only underwent scaling, nor were there differences in the groups treated with aPDT. On the other hand, in a study of patients with HIV [31], there was a beneficial effect of aPDT, with improvements in clinical parameters, and the beneficial effects persisted for up to six months. However, no significant differences in microbial composition were found when compared to the group that received conventional treatment (SRP). More recently, a study has demonstrated promising results in patients with Down syndrome [49]. Therefore, future research may include a broader range of patients with other special needs or specific conditions, such as those in intensive care units.

The variability of results is also evident in studies involving smoking patients. aPDT may contribute to improvements in clinical parameters, but none of the studies showed statistically promising results for therapy in the smoking group [15,16,17]. There were worse clinical outcomes for the smoking group compared to non-smokers, as higher probing depth (PD) and clinical attachment level (CAL) values were observed in smokers at the 3-month follow-up [15]. This indicates uncertainty regarding the effectiveness of the therapy in this patient profile.

The inclusion of studies focusing on patients with systemic conditions and deleterious habits reflects a deliberate effort to address populations that are often underrepresented in systematic reviews. This approach highlights the need to explore the applicability of aPDT in clinically complex scenarios and should serve as a stimulus for future studies targeting these and other systemic complications with potential periodontal repercussions. Thus, when comparing different profiles of clinically complex patients, we observed that the results of the studies do not allow us to prudently state that photodynamic therapy presents fully effective benefits in clinical practice, or even that there is sufficiently robust evidence to support such a conclusion. In this comparison, the results were more favorable for patients with HIV and Down syndrome [31,49]. Nevertheless, it is important to emphasize that there are significant methodological variations among the studies analyzed.

A systematic review reported noteworthy findings. The authors observed that in studies with parallel and split-mouth designs, there were no significant differences in clinical outcomes between the approaches. They also highlighted that an application time of 60 s was more effective in reducing probing depth and improving clinical attachment gain. However, due to the wide variety of protocols used in the studies, it was not possible to conduct a consistent analysis regarding the type of photosensitizer or laser parameters. What could be observed, however, is that all studies employed wavelengths between 660 and 680 nm, which coincide with the absorption peaks of common photosensitizers such as phenothiazine chloride (HELBO^®^) and methylene blue (Periowave^®^ or other preparations) [41]. This suggests that the positive effects of aPDT may be linked to these specific evaluation conditions, although further research is needed. A degree of positive results can be noted when analyzing the findings of systematic reviews [50]. On the other hand, another review indicated that despite aPDT being extensively investigated, evidence of its superior clinical benefits compared to SRP alone remains uncertain. The analyzed data did not show consistent improvements in parameters such as probing depth (PD), bleeding on probing (BOP), and clinical attachment level (CAL) six months after treatment [32]. It is also important to note that all these systematic reviews did not focus exclusively on methylene blue but included various types of photosensitizers, which is the primary focus of this review.

Therefore, when considering systematic reviews (without specifying the type of photosensitizer used), conflicting results have been observed. In one systematic review, the meta-analysis did not demonstrate a statistically significant effect in favor of aPDT [4]. Furthermore, modest statistical gains were observed with the combination of aPDT and scaling and root planing (SRP) [33,35,36,37]. However, it has been questioned whether these findings translate into meaningful clinical relevance [34]. Other systematic reviews have demonstrated that aPDT yields positive results when used as an adjunct to SRP in the treatment of chronic periodontitis [34,36] and aggressive periodontitis [38]. Furthermore, aPDT has shown greater efficacy than systemic antibiotics [35] and has promoted additional clinical improvement in the periodontal treatment of residual pockets [37].

More recently, a systematic review by Alasqah (2024) [33] aimed to specifically evaluate the use of methylene blue-mediated aPDT as an adjunctive approach to periodontal therapy. The review demonstrated that MB-mediated aPDT, when used as an adjunct to mechanical debridement (MD), contributes to improvements in plaque index (PI), probing depth (PD), and bleeding on probing (BOP) in patients with periodontitis. In addition, the meta-analysis revealed statistically significant improvements in plaque index in favor of aPDT.

Unfortunately, some studies employ an inappropriate combination of photosensitizer (PS) and laser wavelength, mismatched with the PS absorption spectrum [25,51,52,53]. The main principle in aPDT is that the absorption peak of FS must be in the same range of the laser wavelength to create a photodynamic reaction (The Grotthuss–Draper law—Principle of Photochemical Activation). That is why blue FS (toluidine and MB) are used with red lasers. It is of utmost importance that the combination of the photosensitizer and the laser wavelength is appropriately applied in clinical trials, and this information should be critically evaluated and reported by journal reviewers [54].

Some of the studies addressed in this review presented satisfactory clinical outcomes in the group of patients who received aPDT, with these benefits typically being short-term, showing improvement within 1 month of follow-up [11]. However, the 6-month follow-up did not show a statistically significant difference between the patients who received aPDT and those who only received scaling and root planing (SRP). This is further compounded by the fact that these studies involved single-session aPDT. Studies demonstrate that increasing the pre-irradiation time and performing multiple sessions of aPDT appear to benefit both clinical and immunological outcomes [23,26].

The lack of consensus on protocols, along with the variability of clinical outcomes among patients with different systemic conditions and habits, such as smoking, may limit the universal applicability of the technique. Future investigations should prioritize controlled and randomized clinical trials that adopt a more rigorous methodological approach, aiming to conduct long-term follow-ups and therapy applications to assess the maintenance of aPDT benefits over time and its true effectiveness in specific patient subgroups.

Despite the promising findings observed in this review, it is essential to acknowledge its methodological limitations, which impose significant constraints on the generalizability of the results. The substantial heterogeneity among the included studies, particularly regarding aPDT application protocols (such as photosensitizer concentration, number and frequency of applications, pre-irradiation time, and light dosimetry parameters), hampers direct comparisons and compromises the standardization of interventions. Moreover, the studied populations exhibited diverse clinical profiles, including patients with various systemic conditions such as diabetes, HIV, and Down syndrome, all of which may influence the response to periodontal treatment and aPDT. These methodological and population-related variations impact the consistency of outcomes and limit the strength of the conclusions concerning the clinical efficacy of the therapy. For these reasons, caution is warranted when interpreting the data and extrapolating the findings to clinical practice. The consolidation of aPDT as an effective adjunctive therapy in the treatment of periodontitis depends on well-designed randomized clinical trials employing standardized protocols, representative samples, and longitudinal follow-up to validate the observed effects.

## 5. Conclusions

This review contributes to the existing literature by synthesizing and critically analyzing recent evidence on the efficacy of MB-mediated aPDT, with particular emphasis on protocol heterogeneity, specific patient profiles, and reported clinical outcomes. Antibacterial photodynamic therapy associated with methylene blue seems to contribute to the clinical improvement of periodontal parameters, particularly PD, BOP, CAL, and PI. However, the heterogeneity of clinical trials creates a duality regarding the role this therapy may play for patients with periodontitis. In the reviewed studies, there is a general trend of absence of statistically significant results between groups over the evaluation periods, or a lack of clinical significance. However, when differences are found, the group that received aPDT tends to show more satisfactory results, highlighting its true additional clinical benefits (PD, BOP, CAL, and PI) in periodontal health. By underscoring the methodological variability and inconsistency of findings across studies, this work highlights the need for caution when generalizing the potential benefits of this adjunctive therapy—especially in populations with systemic conditions, deleterious habits, or complex clinical presentations.

## 6. Future Directions

Based on the findings of this review, aPDT using MB presents promising but still inconclusive implications for the future of periodontics. While several studies report short-term clinical improvements, the variability in patient profiles, application protocols, and follow-up periods raise concerns about reproducibility and generalizability. Future directions in periodontal therapy should focus on the standardization of aPDT protocols, including the definition and control of specific clinical variables such as the wavelength and power of the light source, as well as the method of photosensitizer application. It is also necessary to identify and target specific patient profiles, such as those with systemic conditions (e.g., diabetes and immunosuppression), users of alternative forms of tobacco consumption (e.g., waterpipes and electronic cigarettes), and patients with treatment-resistant periodontitis. These groups should be prioritized in future randomized clinical trials. Such stratification may help determine which subgroups benefit most from antimicrobial photodynamic therapy, enabling a more personalized and effective periodontal approach. Future directions in periodontal therapy should focus on standardizing aPDT protocols, including photosensitizer concentration (with an appropriate light source), irradiation time, and frequency of application (multiple sessions). Moreover, further randomized controlled trials with robust design and long-term molecular and clinical follow-up are essential to establish aPDT as a reliable adjunctive treatment. If such consistency and efficacy can be demonstrated, aPDT may become a valuable tool in the personalized management of periodontal disease, particularly for patients with disabilities, systemic conditions, and users of alternative forms of tobacco consumption (waterpipe or electronic smoking device users) or treatment-resistant profiles.

## Figures and Tables

**Table 1:** Relationship between the number of selected studies and the addressed patient profile.

Patient Profile	Number of Articles (*n*)	Reference
Diabetes	5	[9,10,11,12,13]
Furcation lesion	1	[14]
Smokers	3	[15,16,17]
Periodontitis	9	[18,19,20,21,22,23,24,25,26]
Residual periodontal pockets	3	[27,28,29]
Different patient profiles (Obesity and HIV)	2	[30] (Obesity); [31] (HIV)

**Table 2:** Clinical trials that evaluated the use of photodynamic therapy as an adjunct to scaling and root planing in periodontal treatment using MB as the photosensitizer (PS).

Authors, Year and Participant (*n*)	Wavelength	Laser Parameters	Optic Fiber	Concentration of Dye	Repetition	Main Results
Cunha et al. (2024) (*n* = 38) [9]	650	100 mW/80 s	Optic fiber (d = 600 μm)	10 mg/mL	3 sessions	SRP group presented greater values of PD (*p* < 0.05). There was a significant reduction in TNF-α in crevicular fluid of patients treated by aPDT (*p* < 0.05)
Rodrigues et al. (2023) (*n* = 14) [18]	660	100 mW/0.25 mW/cm^2^/14.94 J/cm^2^/10 s	NR	1%	2 sessions	aPDT promoted better results of PD after 3 months. There was 18% less probability of presenting a final PD > 4 mm compared to SRP.
Cláudio et al. (2021) ** (*n* = 34) [10]	660	157 J/cm^2^/100 mW/50 s	Optic fiber (d = 0.03 cm^2^)	10 mg/mL	3 sessions	aPDT presented a reduction in NRP after 3 and 6 months (*p* < 0.05).
Derikvand et al. (2020) (*n* = 50) [19]	660	150 mW/60 s	NR	100 μg/mL	Single	Reduction in PD at aPDT group after 3 and 6 months, in comparison to SRP group (*p* < 0.01).
Katsikanis et al. (2020) ** (*n* = 21) [16]	670	350 mW/0.445 W/cm^2^/120 s	Diameter—1 cm	1%	3 sessions	Only PI presented statistically significant differences at baseline (*p* = 0.038) in SRP group.
Alahmari et al. (2019) (*n* = 83) [15]	660	150 mW/75 mW/cm^2^/60 s	Optic fiber (d = 600 μm)	0.005%	Single	Only PI in SRP group presented statistically significant differences (*p* < 0.05) after 1 month. PD and CAL were greater in S group When compared to NS group.
Barbosa et al. (2018) (*n* = 12) [11]	660	40 mW/120 s/4.8 J	-	10 mg/mL	Single	There was no difference between groups for PD and CAL (*p* > 0.05). aPDT group presented better results for PI after 1 month and BOP after 6 months.
Andere et al. (2018) (*n* = 36) [20]	660	60 mW/129 J/cm^2^/60 s	Optic fiber	10 mg/mL	Single	Group UPD + CLM + aPDT presented greater CAL values when compared to UPD and UPD + aPDT (*p* < 0.05).
Theodoro et al. (2018) (*n* = 51) [17]	660	100 mW/160 J/cm^2^/48 s	Optic fiber (d= 0.03 cm2)	10 mg/mL	3 sessions	After 6 months, group MTZ + AMX and aPDT presented lower PD, greater CAL and less BOP, but without statistically significant differences between SRP and aPDT.
Vohra et al. (2018) ** (*n* = 52) [30]	670	150 mW/60 s	Optic fiber (d = 0.6 mm)	0.005%	Single	PI was better for SRP group after 1.5 and 3 months (*p* < 0.05).
Al-Askar et al. (2017) (*n* = 70) [12]	670	150 mW/60 s	NR	0.005%	Single	There was no difference between groups and periods. There was no difference in CBL in all groups at 3 and 6 months.
Andrade et al. (2017) (*n* = 28) [21]	660	40 mW/90 s/90 J/cm^2^	Optic Fiber (d = 200 μm)	0.01%	Single	There were no differences between groups. There was a reduction in IL-8 in aPDT group after 3 months (*p* = 0.04).
Pulikkotil et al. (2016) (*n* = 20) [22]	Red LED (628 Hz)	628 Hz/20 s	NR	NR	Single	There was a significant reduction in BOP after 3 months in aPDT group. (*p* < 0.01). There were no differences in A.a. quantification.
Campanile et al. (2015) (*n* = 28) [27]	670	280 mW, ±0.2 dB	Optic fiber	NR	Twice a week	aPDT group presented reduction in PD after 3 and 6 months. There was a reduction in C reactive protein. There were no microbiological differences.
Kolbe et al. (2014) (*n* = 22) [28]	660	0.06 W, 129 J/cm^2^, 60 s	Optic fiber (d = 600 μm)	10 mg/mL	Single	There were no statistically significant differences in clinical parameters. Reduction in Pg., Aa., and inflammatory cytokines.
Franco et al. (2014) ** (*n* = 15) [23]	660	0.06 W/cm^2^, 90 s, 5.4 Jcm^2^	Optic fiber (d = 0.4 mm)	0.01%	Once a week—total of 4 sessions	Reduction in BOP in aPDT group (*p* < 0.05). Increase in RANK/OPG and FGF-2 levels.
Betsy et al. (2014) (*n* = 88) [24]	655	1 W, 0.06 W/cm^2^,60 s	Optic fiber (d = 0.5 mm)	10 mg/mL	Single	Significant reductions in PD, CAL, BOP, PI and GI for aPDT group (*p* < 0.05).
Luchesi et al. (2013) (*n* = 37) [14]	660	0.06 W, 129 J/cm^2^, 60 s	Optic fiber (d = 600 μm)	10 mg/mL	Single	There were no statistically significant differences in clinical parameters. Reduction in Pg., Aa., and inflammatory cytokines up to 6 months.
Dilsiz et al. (2013) (*n* = 24) [25]	808	0.1 W, 6 J, 60 s	Optic fiber (d = 300 μm)	1%	Single	aPDT group presented reduction in PD and CAL after 6 months. (*p* < 0.05).
Campos et al. (2013) (*n* = 13) [29]	660	0.06 W, 129 J/cm^2^, 60 s	Optic fiber (d = 600 μm)	10 mg/mL	Single	aPDT group presented reduction in PD, CAL, and BOP after 6 months.
Noro Filho et al. (2012) (*n* = 12) [31]	660	0.03 W, 0.428 W/cm^2^, 57.14 J/cm^2^, 133 s	Optic fiber (a = 0.07 cm^2^)	0.01%	Single	aPDT presented reduction in PD after 6 months, BOP after 3 and 6 months. There were no differences in microbiological parameters.
Giannelli et al. (2012) (*n* = 26) [26]	635	0.1 W, 120 s (60 s inside + 60 s outside) d = 0.6 mm	Optic fiber (d = 0.6 mm)	0.3%	4 to 10 sessions	aPDT presented reduction in PD, CAL, BOP and spirochetes after 12 months.
Al-Zahrani et al. (2009) (*n* = 45) [13]	670	60 s	NR	0.01%	Single	There were no significant differences.

** = In this article, averages were calculated from the ranges presented; therefore, they are estimated values; aPDT = antimicrobial photodynamic therapy; a = area; AMX = amoxicillin; CBL = crestal bone loss; CLM = clarithromycin; d = diameter; DMT1 = type 1 diabetes mellitus; UPD = ultrasonic periodontal debridement; S = smokers; HbA1c = glycated hemoglobin index; PI = plaque index; GI = gingival index; m = months; MTZ = metronidazole; CAL = Clinical Attachment Level; *n* = number; NRP = number residual pockets; NS = non-smokers; NR = not related; PD = probing depth; Aa. = *Aggregatibacter actinomicetemcomitans*; Pg. = *Porphyromonas gingivalis*; Pi. = *Prevotella intermedia*; Pm = *Parvimonas micra*; Td = *Treponema denticola*; Tf = *Tannerella forsythia*; GR = gingival recession; BOP = bleeding on probing; s = seconds; SRP = scaling and root planing.

**Table 3:** Study evaluation time.

Evaluation Time (Months)	Number of Articles (*n*)	Reference
0 1, 3 and 6	3	[9,11,24]
0 and 3	4	[13,18,23,29]
0, 3 and 6	8	[10,12,14,16,17,20,27,28]
0, 1.5, 3 and 6	2	[19,31]
0, 1 and 3	2	[15,22]
0, 1.5 and 3	1	[30]
0, 3 and 12	1	[21]
0 and 6	1	[25]
0 and 12	1	[26]

**Table 4:** Randomness of the distribution of study groups.

Randomization Process		
Method Used	Number of Articles (*n*)	Reference
Coin toss	3	[15,22,30]
Computer-generated list	8	[13,14,20,25,27,28,29,31]
Computer-generated numbers	1	[9]
Random numbers	1	[24]
Online randomizer	3	[10,17]
Deck of cards	1	[18]
Lottery draw	1	[19]
Randomization chart	1	[16]
Computer software	2	[11,21]
Drawing lots from an opaque bag	1	[12]
Sealed opaque envelopes	1	[26]
Not reported	1	[23]

**Table 5:** Presentation of statistically significant differences according to each variable.

Authors	Patient Profile	PD	BOP (%)	CAL	PI (%)	GI	GR
Cunha et al. (2024) [9]	Periodontitis/Type 1 Diabetes Mellitus	SRP (*p* < 0.05)	NHE	NHE	NHE	-	-
Rodrigues et al. (2023) [18]	Periodontitis	aPDT (*p* = 0.02 at 3 months	-	NHE	-	-	NHE
Cláudio et al. (2021) [10]	Diabetes Mellitus	NHE	NHE	NHE	NHE	-	NHE
Derikvand et al. (2020) [19]	Periodontitis	aPDT (*p* < 0.01) at 3 and 6 months	-	-	NHE	NHE	-
Alahmari et al. (2019) [15]	Smokers	NHE	NHE	NHE	SRP (*p* < 0.01) at 1 month	-	-
Katsikanis et al. (2020) [16]	Moderate smoker	NHE	NHE	NHE	SRP (*p* = 0.038) at baseline	-	-
Barbosa et al. (2018) [11]	Periodontitis/Diabetes Mellitus	NHE	aPDT (*p* = 0.05) at 6 months	NHE	aPDT (*p* = 0.02) only at 1-month follow-up	-	-
Andere et al. (2018) [20]	Periodontitis	aPDT (*p* < 0.05) at 3 months	NHE	NHE	-	-	NHE
Theodoro et al. (2018) [17]	Smokers	NHE	NHE	NHE	-	-	-
Vohra et al. (2018) [30]	Obesity/Periodontitis	NHE	NHE	NHE	SRP (*p* < 0.01) at 1.5 months and 3 months	-	-
Al-Askar et al. (2017) [12]	Pre-diabetes	NHE	NHE	-	NHE	-	-
Andrade et al. (2017) [21]	Periodontitis	NHE	NHE	NHE	NHE	-	-
Pulikkotil et al. (2016) [22]	Periodontitis	NHE	aPDT (*p* < 0.01) at 3 months	NHE	NHE	-	-
Campanile et al. (2015) [27]	Residual pockets	aPDT (*p* = 0.04) at 3 months	NHE	NHE	NHE	NHE	-
Kolbe et al. (2014) [28]	Residual pockets	NHE	NHE	NHE	-	-	-
Franco et al. (2014) [23]	Periodontitis	NHE	aPDT (*p* < 0.05)	NHE	NHE	-	-
Betsy et al. (2014) [24]	Periodontitis	aPDT (*p* < 0.05) at 3 and 6 months	aPDT (*p* < 0.05) at 1 and 3 months	aPDT (*p* < 0.05) at 3 and 6 months	aPDT (*p* < 0.05) at 2 weeks	aPDT (*p* < 0.05) at 1 and 3 months	NHE
Luchesi et al. (2013) [14]	Furcation Class III	NHE	NHE	NHE	NHE	-	-
Dilsiz et al. (2013) [25]	Periodontitis	aPDT (*p* < 0.05) at 6 months	NHE	aPDT (*p* < 0.05) at 6 months	NHE	NHE	-
Campos et al. (2013) [29]	Residual pockets	aPDT (*p* < 0.05) at 3 months	aPDT (*p* < 0.05) at 3 months	aPDT (*p* < 0.05) at 3 months	-	-	-
Noro Filho et al. (2012) [31]	HIV	aPDT (*p* < 0.05) at 6 months	aPDT (*p* < 0.05) at 3 and 6 months	NHE	NHE	-	NHE
Giannelli et al. (2012) [26]	Periodontitis	aPDT (*p* < 0.001) at 12 months	aPDT (*p* < 0.001) at 12 months	aPDT (*p* < 0.001) at 12 months			
Al-Zahrani et al. (2009) [13]	Diabetes Mellitus	NHE	NHE	NHE	NHE	-	-

- = not reported by the study; PD = probing depth; BOP = bleeding on probing; CAL = Clinical Attachment Level; PI = plaque index; GI = gingival index; GR = gingival recession; aPDT = SRP + antimicrobial photodynamic therapy group; SRP = group that received only scaling and root planning; NHE = no statistically significant difference was found between the groups; m = months; *p* < 0.05 = statistically significant difference between the groups.

**Table 6:** Presentation of scientific evidence from systematic reviews.

Authors and Year	Selected Articles and Study Participants (*n*)	Conclusion
Jervøe-Storm et al. (2024) [32]	50 selected articles (*n* = 1407)	The available evidence is quite limited, making it difficult to draw definitive conclusions about the superior clinical benefits of aPDT as an adjunctive therapy in the active treatment or maintenance of periodontitis. Furthermore, the data suggest that the observed improvements may be too small to hold clinical relevance. To enhance the reliability of these findings, it is essential to conduct large, well-designed, and rigorously evaluated randomized controlled trials (RCTs), taking into account the variability of outcomes over time.
Alasqah et al. (2024) [33]	11 selected articles (*n*= 455) ***	Methylene blue-mediated antimicrobial photodynamic therapy (aPDT) resulted in statistically significant improvements in clinical parameters, including plaque index (PI), probing depth (PD), and bleeding on probing (BOP) in patients with periodontitis. However, no significant differences were observed in clinical attachment level (CAL) when compared to conventional treatment alone. Due to the heterogeneity of protocols and methodological limitations of the included studies, the authors recommend cautious interpretation of the findings and emphasize the need for further randomized clinical trials with standardized protocols and long-term follow-up to validate the efficacy of aPDT.
Salvi et al. (2020) [4]	17 selected articles (*n* = 370)	The available evidence on adjunctive therapy with lasers and aPDT is limited to a small number of controlled studies, with notable variability in study designs.
Chambrone; Wang; Romanos, (2018) [34]	26 selected articles (*n* = 686)	aPDT may provide additional clinical benefits in patients with periodontitis and peri-implantitis, particularly in reducing probing depth (PD) and improving clinical attachment level (CAL). However, these improvements were modest (generally less than 1 mm), and the quality of the available evidence was rated as low to moderate. Therefore, the authors recommend cautious interpretation of the findings and emphasize that most clinical recommendations in favor of aPDT are still primarily based on expert opinion. Further studies with greater methodological rigor are needed to definitively validate its efficacy.
Akram et al. (2017) [35]	5 selected articles (*n* = 159)	Meta-analysis demonstrated a statistically significant gain in clinical attachment level (CAL) (WMD = 0.60; 95% CI: 0.25 to 0.95; *p* = 0.001), but not in probing pocket depth (PPD) reduction (WMD = 0.67; 95% CI: –0.36 to 1.71; *p* = 0.204), when comparing aPDT to adjunctive antibiotic therapy at follow-up. Whether aPDT is more effective than adjunctive antibiotic therapy in the treatment of periodontitis remains inconclusive, as the current body of evidence is weak. Caution is warranted in interpreting these findings due to the small sample size and high heterogeneity across studies.
Xue et al. (2017) [36]	11 selected articles (*n* = 243)	aPDT provides short-term clinical benefits in patients with chronic periodontitis, primarily in the reduction in probing depth (PD) and, to a lesser extent, in clinical attachment level (CAL) gain. These effects were statistically significant at 3 months, but were not consistently sustained at 6 months. Furthermore, the benefits were more pronounced in non-smoking patients.
Xue; Zhao, (2017) [37]	4 selected articles (*n* = 62)	aPDT provides additional clinical benefits in the treatment of residual periodontal pockets in patients with chronic periodontitis undergoing supportive periodontal therapy. Pooled data from four randomized clinical trials demonstrated statistically significant reductions in probing depth (MD = 0.69 mm) and gains in clinical attachment level (MD = 0.60 mm) when compared to scaling and root planing (SRP) alone. However, these effects were significant only in non-smoking patients, as studies including smokers did not reveal clinically relevant differences between treatment modalities
Vohra et al. (2016) [38]	7 selected articles (*n* = 218)	In the use of aPDT for the treatment of aggressive periodontitis, the authors concluded that this therapeutic approach may be effective as an adjunct to SRP. However, further randomized clinical trials are needed to confirm these findings.
Javed et al. (2013) [39]	6 selected articles (*n* = 615 and 270 *)	aPDT was analyzed as an adjunct to non-surgical periodontal therapy in immunocompromised patients. After review, only six articles were found, of which only one was a randomized clinical trial; the others were laboratory studies conducted in rats. Various factors, such as smoking and poor oral hygiene, may interfere with the outcomes, making it difficult to assess the effectiveness of this therapy. In conclusion, further studies are needed.
Sgolastra et al. (2013) [40]	7 selected articles (*n* = 261)	After evaluating seven articles, the clinical outcomes were found to be modest, indicating a lack of scientific evidence and the need for further studies to assess the efficacy of aPDT as an adjunct to SRP.
Sgolastra et al. (2013) [41]	14 selected articles (*n* = 389)	A more rigorous systematic review was recently published, including 14 studies, but without promising results. aPDT may have short-term effects, as the evidence does not indicate significant differences after six months. Therefore, the authors recommend conducting additional clinical trials with long-term follow-up.
Atieh, 2010 [42]	4 selected articles (*n* = 161)	The analysis included only four articles, with a post-therapy follow-up of three weeks. The results showed a clinical gain of 0.29 mm in attachment level and a reduction of 0.11 mm in probing depth. The authors concluded that the use of aPDT may be beneficial, but they cautioned about the limitation of the small number of studies included.
Azarpazhooh et al. (2010) [43]	5 selected articles (*n* = 74 and 62 **)	The review included five studies, three of which were similar to Atieh, 2010, [42] without distinguishing the types of periodontitis between them, considering studies with follow-up at 3 and 6 months. The results showed a minimal gain in clinical attachment (0.34 mm) and a reduction in probing depth (0.25 mm). The authors concluded that aPDT was not shown to be more effective.

* = animal studies; ** = sites, *** only including studies that used methylene blue for aPDT.

## Data Availability

Material will be available upon request to interested researchers.
